# Carpal Boss: A Case Series of a Radiological Enigma in Dorsal Wrist Pathology

**DOI:** 10.7759/cureus.68078

**Published:** 2024-08-29

**Authors:** Hejjaji Anand Krishnamurthy, Harshith Gowda, Pooja Basavaradhya Parameshwara, Arjun Raju P, Saitejas Mudunuri, Pratapsingh Parihar

**Affiliations:** 1 Radiology, Tenet Diagnostics, Bangalore, IND; 2 Radiodiagnosis, Jawaharlal Nehru Medical College, Datta Meghe Institute of Higher Education and Research, Wardha, IND

**Keywords:** radiological diagnosis, pseudoarthrosis, osteoarthritic changes, ct scan, mri, carpometacarpal joint, carpal boss

## Abstract

Carpal boss, a relatively rare and often underdiagnosed condition, is characterized by a bony protuberance at the dorsal aspect of the carpometacarpal (CMC) joint. It is commonly misdiagnosed due to its nonspecific clinical presentation, which can mimic other conditions, such as ganglion cysts or fractures. This case series aims to document and analyze the clinical presentations and radiological findings of three patients diagnosed with carpal boss, highlighting the importance of advanced imaging techniques in accurate diagnosis and management. A case series was conducted at Tenet Diagnostics, Bengaluru, Karnataka, involving three patients with dorsal wrist pain and swelling at the CMC joint. All patients underwent clinical evaluation followed by imaging studies using 3T magnetic resonance imaging (MRI) (United Imaging, Shanghai, China) and 32-slice computed tomography (CT) (Siemens Somatom Go; Siemens Healthineers, Munich, Germany). The MRI sequences included T1-weighted and proton density (PD) fat-saturated images, while CT imaging focused on axial and sagittal sections to assess bony structures. All three patients were diagnosed with carpal boss based on imaging findings. MRI revealed hypertrophied bony protuberances at the bases of the second and third metacarpal bones, forming pseudoarthrosis with associated osteoarthritic changes. CT imaging confirmed these findings, providing high-resolution views of the bony abnormalities. Combining MRI and CT was crucial in differentiating carpal boss from other potential diagnoses, such as ganglion cysts or fractures. This case series underscores the importance of advanced imaging modalities, such as MRI and CT, in diagnosing carpal boss. Accurate and early diagnosis can prevent mismanagement and guide appropriate treatment strategies, improving patient outcomes. Increased awareness of carpal boss among clinicians and radiologists is essential for promptly recognizing and managing this condition.

## Introduction

Carpal boss, or carpometacarpal (CMC) bossing, is a condition characterized by a bony protuberance at the dorsal aspect of the CMC joint, typically involving the bases of the second and third metacarpals and adjacent carpal bones. This condition is often underdiagnosed or misdiagnosed due to its relatively low prevalence and the nonspecific nature of its clinical presentation, which can mimic other common wrist conditions, such as ganglion cysts, tendonitis, or fractures [1,2]. First described by Fiolle in 1931, the carpal boss is considered a type of osteoarthritic change, potentially resulting from repetitive strain or trauma to the wrist, leading to the formation of a bony prominence [3]. The condition is most commonly seen in individuals who engage in repetitive wrist activities, such as athletes or manual laborers, although it can also occur idiopathically [4]. The bony protuberance associated with the carpal boss is often associated with pseudoarthrosis, where the hypertrophied bone may articulate abnormally with adjacent bones, leading to pain and functional impairment [5].

Radiological imaging plays a critical role in the diagnosis of carpal boss. While plain radiographs can sometimes identify the bony prominence, advanced imaging techniques, such as magnetic resonance imaging (MRI) and computed tomography (CT), are more effective in providing detailed information about the bony and soft tissue structures involved. MRI is particularly useful for assessing soft tissue changes and bone marrow involvement, while CT offers high-resolution images of the bony protuberances and associated osteoarthritic changes [6]. Despite its significance, the carpal boss remains an under-recognized condition, with many cases being misdiagnosed or overlooked in clinical practice. This underlines the need for increased awareness among clinicians and radiologists to ensure accurate and timely diagnosis, which is essential for appropriate management and prevention of long-term complications [2].

## Case presentation

This was a case series conducted at Tenet Diagnostics, Bengaluru, Karnataka, between January 2023 and June 2024. The primary objective was to analyze the clinical and radiological characteristics of patients diagnosed with carpal boss, a relatively rare condition. By focusing on a series of cases, the study aims to contribute to the existing knowledge base and improve awareness of this condition among clinicians and radiologists. The study included three patients with symptoms of dorsal wrist pain and swelling at the CMC joint. These patients were selected based on their clinical presentation and subsequent confirmation of carpal boss through imaging studies. The inclusion criteria required a clinical suspicion of the carpal boss, followed by radiological confirmation. Patients with other diagnoses or insufficient imaging data were excluded from the study. Imaging techniques are shown in Table 1.

**Table 1:** Imaging techniques

Imaging modality	System	Purpose
Magnetic resonance imaging	Siemens Sempra (1.5T) [7]	Routine imaging sequences
Magnetic resonance imaging	United Imaging (3T) [8]	High-resolution imaging for soft tissue, bone marrow, and pseudoarthrosis evaluation, including T1-weighted and proton density (PD) fat-saturated sequences.
Computed tomography	Siemens Somatom Go (32 Slice) [9]	Evaluation of bony structures, hypertrophied bony protuberances, osteoarthritic changes, and pseudoarthrosis at the carpometacarpal (CMC) joints, including axial and sagittal sections.

Following a detailed clinical examination, markers were placed at the points of interest on the patient's wrist. Both MRI and CT scans were then performed on the affected hand. The imaging procedure aimed to identify and characterize the bony protuberances associated with the carpal boss and assess any associated osteoarthritic changes. Combining MRI and CT allowed for a thorough evaluation of soft tissue and bony structures, facilitating an accurate diagnosis. Radiological data from the MRI and CT scans were collected and analyzed to identify common patterns associated with carpal boss. The data were compared with clinical findings to confirm the diagnosis and assess the severity of the condition. Specific imaging features, such as hypertrophied bony protuberances and pseudoarthrosis, were documented and analyzed. The case series included three patients: two males and one female, aged 19 to 40 years. All patients presented with pain and swelling localized to the dorsum of the wrist, specifically at the CMC joint level. The onset of symptoms varied among the patients, with one reporting a history of a sports injury seven years prior and another presenting with recent symptoms. The clinical presentations were initially suspected to be due to ganglion cysts or fractures, highlighting the diagnostic challenges associated with carpal boss.

Case 1

**Figure 1:**
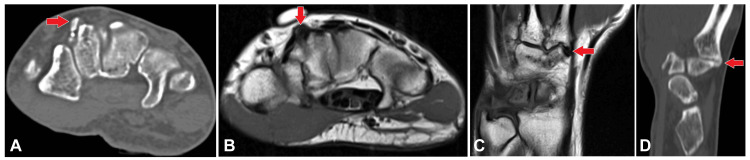
(A)-(D) A marker was placed at the point of interest, and both CT axial imaging and 3T MRI were performed. The provided images include axial and sagittal sections of the MRI (T1-weighted images) and the CT scan. These images reveal hypertrophied bony protuberances at the base of the second metacarpal and trapezoid bones, forming a pseudoarthrosis with mild osteoarthritic changes. The presence of these hypertrophied bony protuberances and associated pseudoarthrosis, with mild osteoarthritic changes, suggests a diagnosis of carpal boss. MRI: Magnetic resonance imaging; CT: Computed tomography

A 29-year-old male presented with persistent dorsal wrist pain and swelling localized to the CMC joint, specifically at the base of the second metacarpal. The patient reported a history of a sports-related wrist injury that occurred seven years prior, resulting in chronic discomfort in the affected area. Initially, the clinical examination suggested the possibility of a ganglion cyst or a fracture due to the nonspecific nature of the symptoms. However, further investigation using advanced imaging modalities provided a more accurate diagnosis. The 3T MRI (United Imaging, Shanghai, China) scan revealed hypertrophied bony growths at the base of the second metacarpal, forming a pseudoarthrosis with mild osteoarthritic changes at the trapezoid bones (Figure 1A). The T1-weighted images were instrumental in identifying these bony protuberances and the associated osteoarthritic changes. The axial 32-slice CT scan (Siemens Somatom Go; Siemens Healthineers, Munich, Germany) corroborated the MRI findings, confirming the presence of a bony protuberance at the base of the second metacarpal, forming a pseudoarthrosis with the trapezoid bones (Figure 1B). These imaging findings were consistent with a diagnosis of carpal boss, differentiating it from the initially suspected ganglion cyst or fracture (Figures 1C-1D).

Case 2

**Figure 2:**
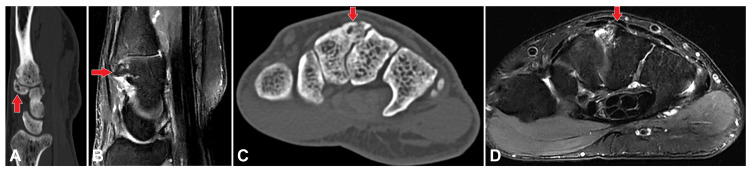
(A)-(D) A marker was placed at the point of interest, and both CT axial imaging and 3T MRI were performed. The provided images include axial and sagittal sections of the MRI (PD fat-saturated images) and the CT scan. These images demonstrate hypertrophied bony protuberances at the base of the third metacarpal and trapezoid bones, forming a pseudoarthrosis with osteoarthritic changes, marrow edema, and subcortical tiny pseudocystic areas. The findings of hypertrophied bony protuberances at the base of the third metacarpal and trapezoid bones, along with the associated pseudoarthrosis, osteoarthritic changes, marrow edema, and subcortical tiny pseudocystic areas, are noted. MRI: Magnetic resonance imaging; CT: Computed tomography; PD: Proton density

A 34-year-old female presented with recent dorsal wrist pain and swelling localized at the base of the third metacarpal. The patient did not report any significant history of trauma or injury. Due to the nonspecific nature of the symptoms, the initial clinical suspicion leaned toward a ganglion cyst. However, the imaging studies provided crucial insights that led to an accurate diagnosis. The 3T MRI scan, particularly the proton density (PD) fat-saturated images, identified hypertrophied bony protuberances at the base of the third metacarpal. The imaging also revealed marrow edema and subcortical tiny pseudocystic areas (Figure 2A), which further guided the diagnostic process. CT imaging was used to validate the MRI findings, providing high-resolution images of the bony structures. The CT scans confirmed the presence of pseudoarthrosis with osteoarthritic changes at the third metacarpal-trapezoid joint (Figure 2B). The combination of MRI and CT imaging solidified the diagnosis of carpal boss, ruling out other potential conditions, like ganglion cysts (Figures 2C-2D).

Case 3

**Figure 3:**
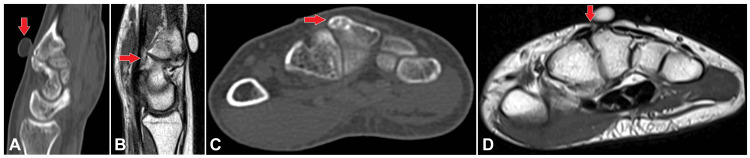
(A)-(D) A marker was placed at the point of interest, and both CT axial imaging and 3T MRI were performed. The provided images include axial and sagittal sections of the MRI (T1-weighted images) and the CT scan. These images reveal a hypertrophied bony protuberance on the dorsal surface of the base of the third metacarpal, with pseudoarthrosis involving the capitate. Additionally, a few tiny cysts are observed at the site of the pseudoarthrosis, accompanied by minimal marrow edema. The findings of the hypertrophied bony protuberance and associated pseudoarthrosis with the capitate are consistent with a diagnosis of carpal boss. MRI: Magnetic resonance imaging; CT: Computed tomography

A 40-year-old male with a history of manual labor presented with progressive dorsal wrist pain and a palpable swelling at the base of the third metacarpal. Over the past year, the patient experienced worsening symptoms, prompting further evaluation. The initial clinical examination did not conclusively identify the underlying cause, leading to the decision to proceed with advanced imaging. The 3T MRI scan, particularly the T1-weighted images, revealed a hypertrophied bony protuberance on the dorsal surface of the base of the third metacarpal (Figure 3A). The images also showed minimal marrow edema and tiny cysts at the site of pseudoarthrosis, which were crucial in diagnosing the condition. CT imaging provided detailed visualization of the bony structures, confirming the presence of pseudoarthrosis between the hypertrophied bony protuberance and the capitate bone (Figure 3B). These findings were consistent with a diagnosis of carpal boss, emphasizing the importance of combining MRI and CT imaging in accurately identifying the condition (Figures 3C-3D).

Following the diagnosis, the patients were informed about the nature of their condition. Treatment options, including conservative management and surgical intervention, were discussed based on the severity of symptoms and the extent of bony changes observed on imaging. All patients opted for conservative management initially, with plans for follow-up to monitor symptoms and evaluate the need for surgical intervention. The results of this case series emphasize the importance of advanced imaging techniques in diagnosing carpal boss. MRI provided crucial information about soft tissue involvement and bone marrow changes, while CT imaging confirmed the presence of bony protuberances and pseudoarthrosis. The combination of these imaging modalities facilitated an accurate diagnosis, underscoring the role of radiological evaluation in managing this uncommon condition.

## Discussion

Carpal boss, also known as CMC bossing, is a relatively rare condition characterized by the presence of a bony protuberance at the dorsal aspect of the CMC joint, most commonly at the second or third metacarpal bases [10]. This condition is often underdiagnosed due to its nonspecific clinical presentation, which can mimic more common conditions, such as ganglion cysts, fractures, or tenosynovitis. The cases presented in this series highlight the diagnostic challenges associated with carpal boss and underscore the critical role of advanced imaging techniques in its diagnosis. The clinical symptoms of carpal boss, including dorsal wrist pain and swelling, are nonspecific and often lead to misdiagnosis. In this series, all patients presented with these symptoms, initially leading to clinical suspicions of ganglion cysts or fractures. This aligns with findings from other studies, where carpal boss is frequently misdiagnosed due to its rarity and overlapping symptoms with other wrist pathologies [11,12]. The delay in accurate diagnosis can lead to prolonged symptoms and inappropriate treatment, underscoring the importance of considering carpal boss in the differential diagnosis of dorsal wrist pain.

Imaging is pivotal in diagnosing carpal boss, especially when clinical findings are inconclusive. MRI and CT scans were utilized in our series to confirm the diagnosis. MRI was particularly useful in assessing soft tissue involvement, bone marrow changes, and the presence of pseudoarthrosis. T1-weighted and PD fat-saturated MRI sequences provided detailed images, demonstrating bony protuberances and associated changes. These findings are consistent with previous studies that emphasize the utility of MRI in diagnosing carpal boss, particularly in cases where there is a suspicion of associated soft tissue or marrow involvement [13,14]. CT imaging further complemented MRI findings by providing high-resolution images of the bony structures. The axial sections obtained from CT scans revealed hypertrophied bony protuberances and pseudoarthrosis, characteristic of the carpal boss. This is in line with other reports that highlight the value of CT imaging in confirming the diagnosis by providing detailed visualization of the bony anatomy [15,16]. Our study's combination of MRI and CT facilitated an accurate and comprehensive evaluation of the condition, ensuring that the diagnosis was not missed. Once diagnosed, the management of carpal boss typically involves conservative approaches, such as rest, splinting, and anti-inflammatory medications, particularly in patients with mild symptoms. However, for those with persistent or severe symptoms, surgical excision of the bony protuberance may be considered [2]. In our series, all patients opted for conservative management initially, with plans for follow-up to assess symptom progression. This approach is consistent with the literature, where conservative management is often the first line of treatment, with surgery reserved for refractory cases [11].

## Conclusions

The cases presented in this series highlight the importance of considering carpal boss as a differential diagnosis in patients presenting with dorsal wrist pain, particularly when the symptoms are chronic or there is a history of trauma. Although relatively rare, carpal boss can significantly impact a patient's quality of life if not accurately diagnosed and managed. Advanced imaging techniques, especially CT and MRI, are crucial in identifying the characteristic bony protuberances and associated changes that define this condition. Early recognition and diagnosis of carpal boss allow for more effective management strategies, including conservative treatment or surgical intervention, depending on the severity of the symptoms and the patient's functional needs. Ultimately, increasing awareness and understanding of carpal boss among clinicians and radiologists can improve patient outcomes and reduce misdiagnoses or unnecessary treatments.
